# Risk stratification of childhood cancer survivors necessary for evidence-based clinical long-term follow-up

**DOI:** 10.1038/bjc.2017.347

**Published:** 2017-10-24

**Authors:** Clare Frobisher, Adam Glaser, Gill A Levitt, David J Cutter, David L Winter, Emma R Lancashire, Kevin C Oeffinger, Joyeeta Guha, Julie Kelly, Raoul C Reulen, Michael M Hawkins

**Affiliations:** 1 Centre for Childhood Cancer Survivor Studies, Institute of Applied Health Research, Robert Aitken Building, University of Birmingham, Birmingham B15 2TY, UK; 2 Leeds Institute of Cancer and Pathology, Clinical Sciences Building, University of Leeds, St James’s University Hospital, Leeds LS9 7TF, UK; 3 Department of Haematology/Oncology, Great Ormond Street Hospital for Children NHS Trust, Great Ormond Street, London WC1N 3JN, UK; 4 Nuffield Department of Population Health, Clinical Trial Service Unit, Richard Doll Building, Old Road Campus, University of Oxford, Oxford OX3 7LF, UK; 5 Departments of Paediatrics and Medicine, Memorial Sloan-Kettering Cancer Centre, 300 East 66th Street, New York, NY 10065, USA; 6 Public Health England, Birmingham And The Black Country Area Team, St Chads Court, 213 Hagley Road, Edgbaston, Birmingham B16 9RG, UK

**Keywords:** clinical follow-up, childhood cancer, risk stratification, adverse health outcomes

## Abstract

**Background:**

Reorganisation of clinical follow-up care in England was proposed by the National Cancer Survivorship Initiative (NCSI), based on cancer type and treatment, ranging from Level 1 (supported self-management) to Level 3 (consultant-led care). The objective of this study was to provide an investigation of the risks of serious adverse health-outcomes associated with NCSI Levels of clinical care using a large population-based cohort of childhood cancer survivors.

**Methods:**

The British Childhood Cancer Survivor Study (BCCSS) was used to investigate risks of specific causes of death, subsequent primary neoplasms (SPNs) and non-fatal non-neoplastic outcomes by NCSI Level.

**Results:**

Cumulative (excess) risks of specified adverse outcomes by 45 years from diagnosis among non-leukaemic survivors assigned to NCSI Levels 1, 2 and 3 were for: SPNs—5% (two-fold expected), 14% (four-fold expected) and 21% (eight-fold expected); non-neoplastic death—2% (two-fold expected), 4% (three-fold expected) and 8% (seven-fold expected); non-fatal non-neoplastic condition—14%, 27% and 40%, respectively. Consequently overall cumulative risks of any adverse health outcome were 21%, 45% and 69%, respectively.

**Conclusions:**

Despite its simplicity the risk stratification tool provides clear and strong discrimination between survivors assigned to different NCSI Levels in terms of long-term cumulative and excess risks of serious adverse outcomes.

Comprehensive reorganisation of clinical follow-up care for cancer survivors was proposed by the National Cancer Survivorship Initiative (NCSI) throughout the National Health Service (NHS) in England (NHS Improvement, 2011a, 2011b; Glaser *et al*, 2013). A prior investigation into the follow-up practices in Children’s Cancer and Leukaemia Group (CCLG) Centres indicated wide variations in the proportion of childhood cancer survivors under hospital follow-up beyond 5 years from diagnosis (Taylor *et al*, 2004). The NCSI and the Independent Cancer Taskforce proposed that the level of clinical follow-up care offered to each survivor should be evidence-based and correspond to their risk of serious adverse health outcomes (NHS Improvement, 2011a, 2011b; Glaser *et al*, 2013; Independent Cancer Taskforce, 2015). The NCSI proposed a system of risk stratification principally based on cancer type and treatment received and ranges from Level 1 (supported self-management) to Level 3 (multidisciplinary consultant-led clinical care) (NHS Improvement, 2011a, 2011b; Glaser *et al*, 2013). It is a development of our previously proposed methodology for stratifying survivors into three levels of clinical follow-up care (Wallace *et al*, 2001). So far, there have only been a few small-scale studies assessing the risks of adverse health outcomes associated with using this risk stratification tool, which in aggregate relate to just over 900 survivors (Absolom *et al*, 2006; Eiser *et al*, 2006; Michel *et al*, 2009; Edgar *et al*, 2013).

This NCSI risk stratification tool presented here is simple and does not require detailed information on cumulative doses of individual cytotoxic drugs or radiation doses to major organs. This has considerable advantages as such details may not be readily available, particularly if treatments were given decades ago.

Levels 1 and 2 of clinical care proposed by the NCSI have substantially lower frequencies of hospital attendance compared with Level 3. It is essential to investigate whether individuals proposed to be followed-up at Levels 1 or 2 experience an increased risk of serious adverse health-outcomes, which could potentially be avoided, or ameliorated, with appropriate hospital follow-up.

There is a pressing need to reliably quantify the overall risks of adverse health-outcomes associated with the NCSI system of risk stratification, formally introduced in 2011 (NHS Improvement, 2011a, 2011b), because it is in increasingly widespread use in the NHS for childhood cancer survivors. Such usage results from the widespread participation of CCLG Centres in the development work of the NCSI. Data from the British Childhood Cancer Survivor Study (BCCSS) provide an unrivalled opportunity to reliably estimate these risks, as there has been no previous large-scale investigation of the serious health risks associated with the NCSI Levels of care in the long-term, and to the best of our knowledge this is the first risk stratification tool that has been proposed for general clinical use for childhood cancer survivors. We provide a large-scale population-based investigation of the risks of serious adverse health-outcomes associated with the NCSI Levels of clinical care up to 45 years from diagnosis using data from the BCCSS.

## Materials and methods

### Sources of data

1. The BCCSS population-based cohort was used to assess the risks of specific causes of death and subsequent primary neoplasms (SPNs). The BCCSS cohort consists of 17 981 individuals who were diagnosed with cancer, when aged 0–14 years inclusive, between 1940 and 1991, in Britain, and who survived at least 5 years. Information on exposure to initial radiotherapy and chemotherapy was available for the entire cohort in the form YES/NO/NO RECORD. Further details including objectives, methods and response rates are available elsewhere (Hawkins *et al*, 2008). Approval for the study was obtained from the appropriate Research Ethics Committees and the Confidentiality Advisory Group consented to processing identified data without individual patient consent.
2. Between 2001 and 2006 a questionnaire addressing adverse health and social outcomes was sent to those 14 836 BCCSS survivors who were aged at least 16 years via their primary care physician (for the questionnaire see: http://www.birmingham.ac.uk/research/activity/mds/projects/HaPS/PHEB/CCCSS/bccss/documents.aspx). In total 10 483 (71%) questionnaires were completed and returned by December 31, 2006. These questionnaires were used to assess risks of specific non-fatal non-neoplastic adverse health-outcomes. We focused on physical conditions graded 3 or 4 using the Common Terminology Criteria for Adverse Events (CTCAE) version 3 (Cancer Therapy Evaluation Programme, 2006). Such conditions are considered severe, life threatening or disabling.
3. Records of 2844 individuals included within the BCCSS who were treated within one of the national Medical Research Council (MRC) randomised trials into treatment of acute lymphoblastic leukaemia (ALL) were used to investigate risks of adverse health outcomes in relation to treatment received, based on the assumption that treatment was delivered, as prescribed in protocol.

### Risk stratification

**Figure 1:**
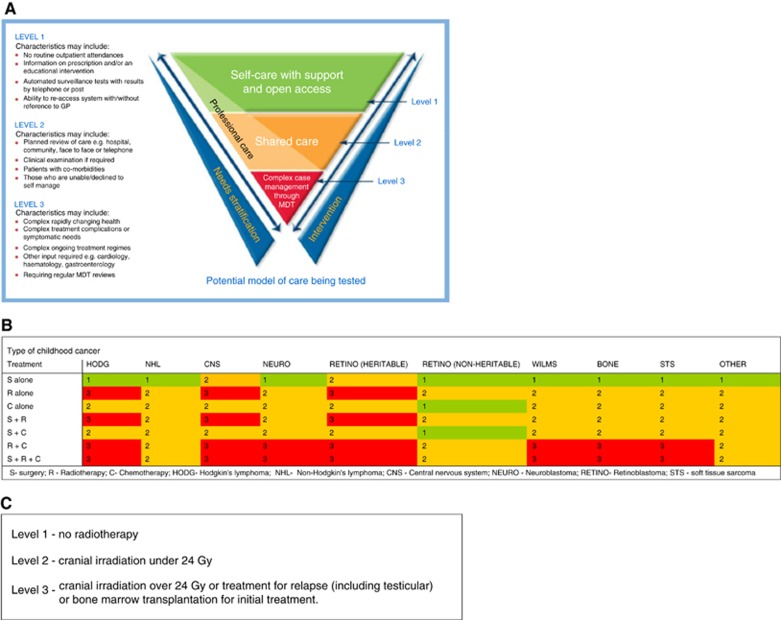
**NCSI Levels of follow-up care.** (**A**) The NCSI clinical levels of care (NHS Improvement, 2011a). (**B**) NCSI Levels of clinical follow-up defined in terms of type of childhood cancer and treatment received. Abbreviations: S=surgery; R=Radiotherapy; C=Chemotherapy; HODG=Hodgkin's lymphoma; NHL=Non-Hodgkin's lymphoma; CNS=Central Nervous System; NEURO=Neuroblastoma; RETINO=Retinoblastoma; STS=soft tissue sarcoma. (**C**) NCSI Levels of clinical follow-up for ALL survivors. Note: There were insufficient survivors of other specific types of leukaemia for meaningful analysis and therefore such survivors are not considered further within this study.

The NCSI clinical Levels of care are described in Figure 1A (further details in Supplementary Appendix 1). For the purposes of the present investigation risk stratification, based on childhood cancer type and its treatment, was undertaken as described in Figure 1B for all childhood cancers except leukaemia and for individuals who were diagnosed with ALL and treated within an MRC trial, as described in Figure 1C.

### Ascertainment and grading of adverse health events

Ascertainment of causes of deaths and incident SPNs within the BCCSS was entirely population-based and achieved by individual patient electronic record linkage via the NHS Information Centre. For each death, we obtained the death certificate and underlying cause of death as coded by the Office for National Statistics. Potential SPNs were confirmed by reviewing relevant diagnostic reports, particularly histopathology reports. Further details are available in our most recent detailed investigations of causes of death (Reulen *et al*, 2010; Fidler *et al*, 2016) and SPNs (Reulen *et al*, 2011) in our cohort.

Occurrence of non-fatal non-neoplastic adverse health-outcomes were ascertained through the BCCSS questionnaire. Each non-fatal non-neoplastic condition was graded using the CTCAE version 3 (Cancer Therapy Evaluation Programme, 2006) by three authors ERL and DLW in collaboration with KCO of the North American Childhood Cancer Survivor Study (CCSS) to ensure comparability with the CCSS. This version of the CTCAE (Cancer Therapy Evaluation Programme, 2006) was used because all related published studies used this version and such standardisation facilitates making satisfactory comparisons. Only events graded 3 or 4 were included here. In addition to indicating the condition, the survivor provided the diagnosis date/age. Potentially treatment-related non-fatal non-neoplastic events were grouped into 10 specific categories as defined by the CTCAE (vision; hearing; speech; circulatory; pulmonary; gastrointestinal; renal; musculoskeletal; neurological; endocrine), plus an overall category for any of these events.

### Statistical methods

Risks of three outcomes were investigated: SPNs; fatal non-neoplastic conditions; non-fatal non-neoplastic conditions. Time at risk for each of these outcomes began at five years subsequent to first primary neoplasm (FPN) diagnosis.

End of period of risk depended on the adverse outcome being analysed. For SPNs/specific fatal non-neoplastic conditions, exit from risk was the date associated with the first of the following events: diagnosis of a SPN/death from specific cause, loss to follow-up, death from other cause or study end-date (the median questionnaire completion date). For non-fatal non-neoplastic conditions, exit from risk was the date of diagnosis of the condition, otherwise the questionnaire completion date.

Survivors were stratified according to their NCSI Levels of clinical care and the cumulative incidence by period of follow-up from diagnosis for each adverse health outcome was estimated. For the estimation of cumulative incidence of a SPN and specific fatal non-neoplastic conditions, other deaths were treated as competing risks (Gooley *et al*, 1999; Coviello and Bogges, 2004). For non-fatal non-neoplastic conditions, the complement of the Kaplan–Meier estimate (1-KM) was used to estimate the cumulative risk. Log rank tests were used to investigate for heterogeneity and trend in cumulative risk.

Standardised incidence ratios (SIRs) and standardised mortality ratios (SMRs) were calculated as the ratio of observed (O) to expected (E) numbers of relevant events (O/E). Expected numbers were estimated by accumulating person years at risk within specific gender and five-year age and one-year calendar period strata and multiplying by gender, age and calendar period specific neoplasm and death rates in the general population of England and Wales. Poisson regression was used to test for heterogeneity and linear trend in SIRs and SMRs across NCSI Levels.

All analyses were carried out using Stata statistical software (version 13; Stata Corp., College Station, TX, USA). Statistical significance was taken at the 5% level, with two-sided tests.

## Results

### Risks after all childhood cancers except leukaemia

#### Risk of subsequent primary neoplasms

**Figure 2:**
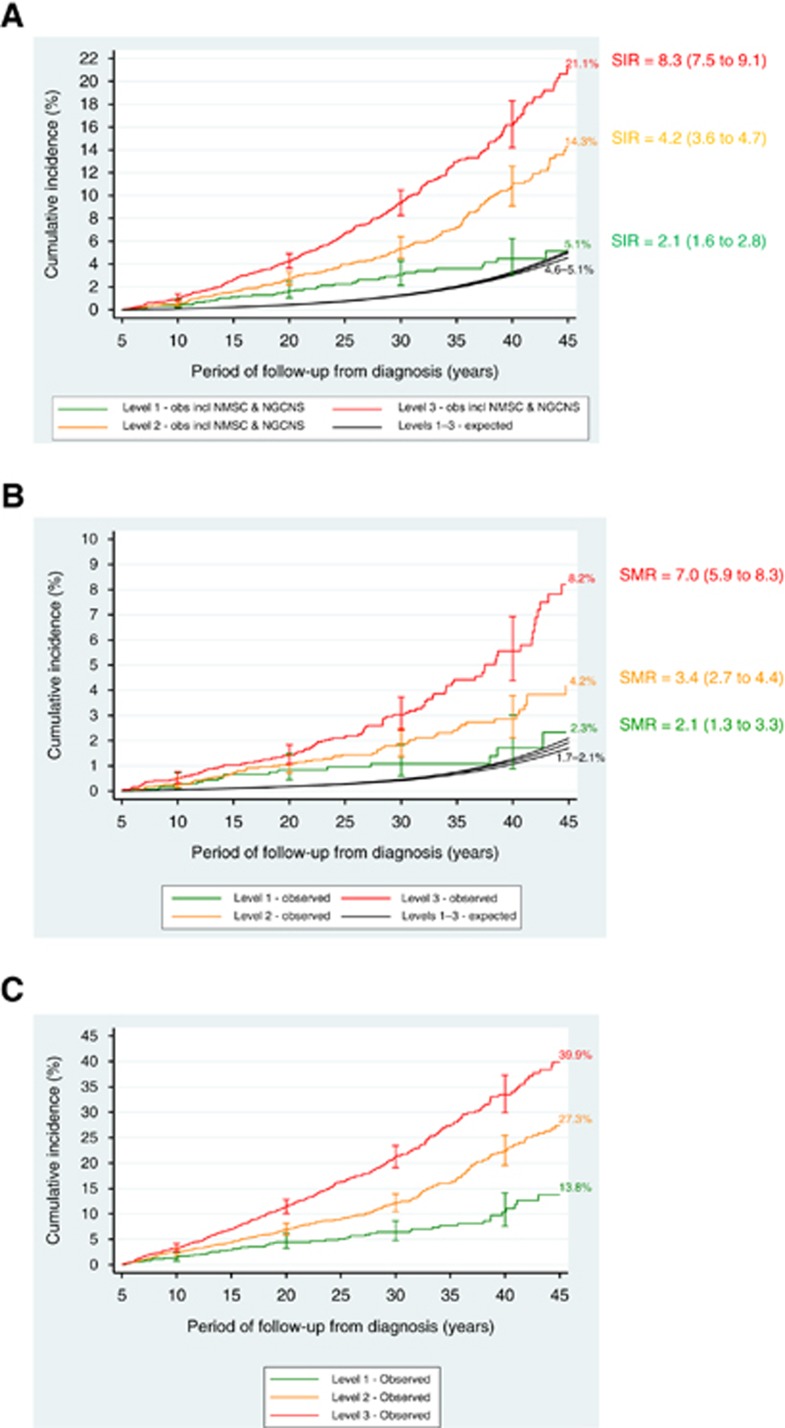
**Risks of adverse health events after all childhood cancers except leukaemia.** (**A**) Observed and expected risks of any subsequent primary neoplasm after all childhood cancers except leukaemia. Abbreviations: incl=including; NGCNS=Non-glioma central nervous system tumours; NMSC=non-melanoma skin cancer; obs=observed. The cross bars are 95% CI. Log-rank test for equality of observed risks yields *P*<0.0001. (**B**) Observed and expected risks of any fatal non-neoplastic event after all childhood cancers except leukaemia. Log-rank test for equality of observed risks yields *P*<0.00005. (**C**) Observed risks of any non-fatal non-neoplastic condition after all childhood cancers except leukaemia. Log-rank test for equality of observed risks yields *P*<0.00005.

Observed cumulative risks (95% CI) by 45 years from diagnosis among survivors assigned to Levels 1, 2 and 3 were 5% (95% CI: 3–7%), 14% (12–17%) and 21% (18–24%), respectively (test for trend *P*<0.0001) (Figure 2A). Corresponding SIRs (95% CI) were 2 (1.6–2.8), 4 (3.6–4.7) and 8-fold expected (7.5–9.1), respectively (test for trend *P*<0.001). Cumulative risks and SIRs were estimated for all SPNs excluding non-melanoma skin cancers (NMSC) and non-glioma central nervous system neoplasms (NGCNS) in Table 1, and gave broadly similar results.

Table 1 also gives the observed cumulative risks by 45 years from diagnosis, and the corresponding SIRs, for specific types of SPNs among survivors assigned to Levels 1, 2 and 3. For digestive, bone, connective and soft tissue, breast and thyroid cancers and for NMSC, glioma CNS neoplasm and NGCNS there was evidence that the SIR increased with increased NCSI Level.

#### Risk of fatal non-neoplastic conditions

Observed cumulative risks (95% CI) by 45 years from diagnosis among survivors assigned to Levels 1, 2 and 3 were 2% (95% CI: 1.1–4.4%), 4% (3.0–5.7%) and 8% (6.3–10.5%), respectively (test for trend *P*<0.00005) (Figure 2B). The corresponding SMRs (95% CI) were 2 (1.3–3.3), 3 (2.7–4.4) and 7-fold expected (5.9–8.3), respectively (test for trend *P*<0.001).

Table 2 (upper half) gives the observed and expected numbers of specific categories of non-neoplastic causes of death across different NCSI Levels, together with the cumulative risks by 45 years from diagnosis. There was evidence of an increase in the SMRs with increased NCSI Level from Level 1 to Level 3 for deaths from circulatory, cerebrovascular, pulmonary and neurological causes.

Among NCSI Level 1 survivors restricting attention to those causes with more than five observed deaths, there was a five-fold excess of deaths from pulmonary causes. Among NCSI Level 2 survivors, restricting attention to those causes with more than five observed deaths, there was a four-fold excess in the number of deaths observed from cardiac, cerebrovascular, pulmonary and neurological causes. Classification to NCSI Level (Figure 1B and C) used only limited information in the BCCSS computer record, before accessing available medical records. Detailed examination of the medical history of these cases, reviewed by physicians, suggested that a greater degree of routine follow-up would not have been helpful in preventing or delaying the excess deaths (data not published due to the potentially individually identifiable nature of the data).

#### Risk of non-fatal non-neoplastic conditions

Figure 2C provides cumulative risks of a non-fatal non-neoplastic (CTCAE grade 3 or 4) condition corresponding to any of the 10 categories specified above, among survivors assigned to the NCSI Levels. The most common conditions diagnosed subsequent to 5-year survival were cardiovascular (19%), endocrine (18%) and pulmonary (14%), together accounting for 51% of all conditions diagnosed. By 45 years from diagnosis the cumulative risk (95% CI) of any potentially treatment-related non-fatal non-neoplastic condition for survivors assigned to NCSI Levels 1, 2 and 3 were 14% (95% CI: 10–19%), 27% (24–31%) and 40% (35–45%), respectively (test for trend, *P*<0.00005).

Table 2 (lower half) gives the cumulative risks of specific potentially treatment-related non-fatal non-neoplastic conditions among survivors by NCSI Level. The cumulative risk of each specific condition increased with increased NCSI Level (all *P*<0.01), with the exception of gastrointestinal and musculoskeletal conditions.

### Risks after acute lymphoblastic leukaemia

#### Risk of SPNs

**Figure 3:**
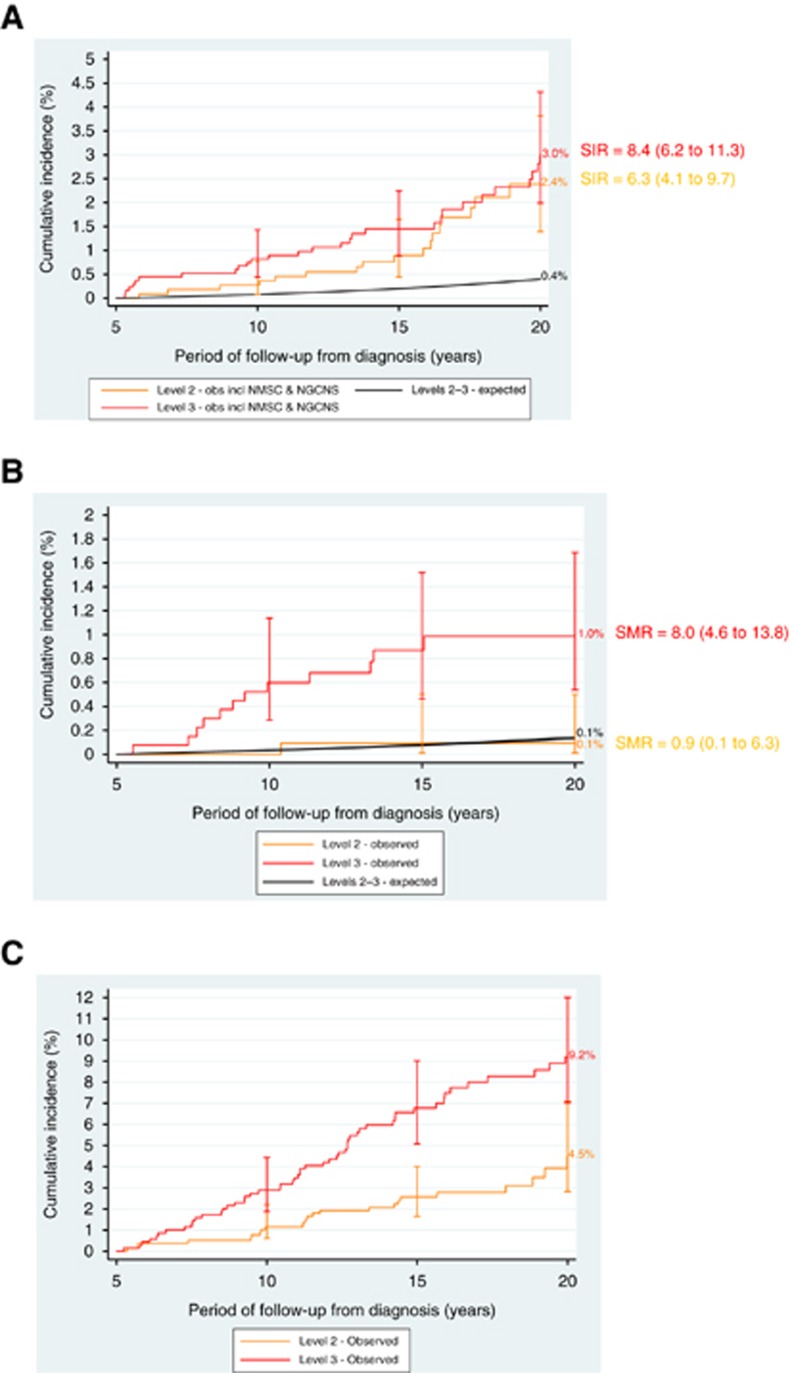
**Risks of adverse health events after acute lymphoblastic leukaemia.** (**A**) Observed and expected risks of any subsequent primary neoplasm after acute lymphoblastic leukaemia. incl=including; NGCNS=Non-glioma central nervous system tumours; NMSC=non-melanoma skin cancer; obs=observed. The cross bars are 95% CI. Log-rank test for equality of observed risks comparing Levels 2 and 3 yields *P*=0.2052. (**B**) Observed and expected risks of any fatal non-neoplastic event after acute lymphoblastic leukaemia. Log-rank test for equality of observed risks comparing Levels 2 and 3 yields *P*=0.0018. (**C**) Observed risks of any non-fatal non-neoplastic condition after acute lymphoblastic leukaemia. Log-rank test for equality of observed risks comparing Levels 2 and 3 yields *P*=0.0002.

Figure 3A provides observed and expected cumulative risks, with corresponding SIRs, of any SPN for survivors assigned to NCSI Levels 2 and 3. Insufficient survivors were classified to Level 1 to reliably estimate risks. Observed cumulative risks (95% CI) by 20 years from diagnosis among survivors assigned to Levels 2 and 3 were 2% (95% CI: 1.4–3.8%) and 3% (2.0–4.3%), respectively (*P*=0.205). The corresponding SIRs (95% CI) were 6 (4.1–9.7) and 8-fold expected (6.2–11.3), respectively (*P*=0.295). Exclusion of NMSC and NGCNS had little impact.

#### Risk of fatal non-neoplastic conditions

Observed cumulative risks (95% CI) by 20 years from diagnosis among survivors assigned to Levels 2 and 3 were 0.1% (95% CI: 0.01–0.5%) and 1% (0.5–1.7%), respectively (*P*<0.01) (Figure 3B). The corresponding SMRs (95% CI) for Levels 2 and 3 were 1 (0.1–6.3) and 8-fold expected (4.6–13.8), respectively (*P*<0.01).

#### Risk of non-fatal non-neoplastic conditions

By 20 years from diagnosis the cumulative risk (95% CI) of any non-fatal non-neoplastic condition for survivors assigned to Levels 2 and 3 were 4% (95% CI: 2.8–7.1%) and 9% (7.0–12.0%), respectively (*P*<0.001) (Figure 3C).

### Risk of SPNs, fatal and non-fatal non-neoplastic conditions among survivors of specific types of cancer assigned to particular NCSI Levels

For evidence-based long-term clinical follow-up the most practically useful risk stratification information relates to the risk of specific adverse outcomes among individuals with a specified type of cancer and NCSI Level. Supplementary Appendix 2 (Supplementary Table 1) provides cumulative and excess risks by 25 years from diagnosis for survivors diagnosed with each specific cancer type (except ALL) and assigned to a particular NCSI Level for which there were at least 100 survivors still at risk at 25 years after diagnosis. After ALL the corresponding interval was 20 years.

#### SPNs

Among survivors assigned to NCSI Levels 1, 2 and 3 the cumulative risks by 25 years from diagnosis (20 years for ALL) were <1%, between 1% and 4%, and between 2% and 13%, respectively (Supplementary Appendix 2). The corresponding SIRs were <2-fold expected, between 2 and 5-fold expected, and between 3 and 17-fold expected, respectively. Among survivors of each specific cancer, both cumulative and excess risks increased with increased NCSI Level, but the risks varied strongly with cancer type.

#### Fatal potentially treatment related non-neoplastic conditions

Among survivors assigned to NCSI Levels 1, 2 and 3 the cumulative risks by 25 years from diagnosis (20 years for ALL) were <1%, between <1% and 2%, and between 1% and 3%, respectively. The corresponding SMRs were as expected, between 1 and 6-fold expected, and between 2 and 9-fold expected, respectively. Again, both cumulative and excess risks increased with increased NCSI Level after specific cancers, but varied importantly by cancer type.

#### Non-fatal potentially treatment related non-neoplastic conditions

The cumulative risks were 5%, between 5% and 12%, and between 7% and 19% by 25 years from diagnosis (20 years for ALL) among survivors assigned to NCSI Levels 1, 2 and 3, respectively.

### Potential impact of missing treatment information

To assign individuals to a NCSI Level we needed the treatment information specified in Figure 1B and C. We have the advantage of the outcomes relating to SPN and death for the entire cohort irrespective of whether treatment information is missing or available. Therefore we can investigate cumulative risks of such outcomes among those with/without sufficient treatment information for risk stratification for evidence of heterogeneity. Similarly among those who returned questionnaires we can compare the risks of non-fatal non-neoplastic outcomes between those with/without sufficient treatment information for risk stratification. Among the entire cohort of 13 130, 5-year survivors of all childhood cancer except leukaemia, 8675 (66%) had sufficient treatment available to assign to Levels 1, 2 or 3. In Supplementary Appendix 3 we investigate cumulative risk of SPN, death and non-fatal non-neoplastic outcomes between those with/without sufficient treatment information. In Supplementary Appendix 4 we undertake a similar investigation in relation to survivors of ALL. There was no evidence of important impact, except that the cumulative risk of SPNs among non-leukaemic survivors was higher among those with sufficient treatment information.

## Discussion

This study provides the first large-scale investigation of the long-term (up to 45 years from diagnosis) risks of serious adverse health-outcomes associated with a simple risk stratification tool which is already being used on an increasingly widespread basis within CCLG Centres throughout the UK. The study has the additional advantage of being population-based. The levels of clinical follow-up care proposed for childhood cancer survivors result from the NCSI (NHS Improvement, 2011a, 2011b) and are a development of a previously proposed risk stratification tool (Wallace *et al*, 2001).

By 45 years from diagnosis, cumulative risks of developing any SPN, dying of any non-neoplastic cause or being diagnosed with any potentially treatment-related non-fatal non-neoplastic condition among survivors assigned to Levels 1, 2 or 3 were 21%, 45% and 69% (Table 3). Excess risks also increased with increasing NCSI Levels 1, 2 and 3: for SPNs two-fold, four-fold and eight-fold expected, respectively; for non-neoplastic deaths two-fold, three-fold and seven-fold expected, respectively.

The risk stratification tool presented above, or an earlier version (Wallace *et al*, 2001), has been investigated in terms of the risks of adverse health-outcomes associated with Levels 1, 2 and 3 in only three previous studies, which included in aggregate just over 900 survivors (Absolom *et al*, 2006; Eiser *et al*, 2006; Michel *et al*, 2009; Edgar *et al*, 2013). The largest of these studies was undertaken by Edgar *et al* (2013) based on 607 (5-year) survivors originally diagnosed with cancer before aged 19 years between 1971 and 2004 at a single institution in Scotland. These investigators reported that the prevalence of adverse health-outcomes increased from 12% to 36% to 65% for Levels 1, 2 and 3, respectively. Restricted to grades ⩾3 in CTCAE version 3 (Cancer Therapy Evaluation Programme, 2006), the corresponding prevalences were 1%, 11% and 39%, respectively. This is much lower than observed in our study and reflects the fact that the percentage of 5-year survivors with a current age beyond 25 years for Levels 1, 2 and 3 were 7%, 27% and 29%, respectively. The BCCSS is a mature cohort and the age distributions between the NCSI Levels do not vary much. From the analysis of all non-fatal non-neoplastic outcomes, 81%, 79% and 80% of the survivors were aged beyond 25 years for Levels 1, 2 and 3. Childhood cancer survivors experience elevated risks of adverse health-outcomes into middle age and beyond, and have an accelerated risk of events over that normally seen with ageing (Oeffinger *et al*, 2006; Reulen *et al*, 2010, 2011; Armstrong *et al*, 2014; Fidler *et al*, 2016). In the current study those assigned to NCSI Levels 1, 2 and 3 were found to experience an increasing risk of a severe adverse health-outcome by 45 years from FPN diagnosis—21%, 45% and 69%, respectively.

The second largest study was based on 198 survivors recruited from one paediatric and one adult follow-up clinic in England, they found that Level 3 survivors, as defined by Wallace *et al* (2001) reported more short-term symptoms attributable to cancer treatment (for example, pain, fatigue, breathlessness) than Level 2 survivors, and Level 1 reported none (Absolom *et al*, 2006; Eiser *et al*, 2006). Also Level 3 survivors reported more late effects (for example, infertility, cardiac dysfunction, second cancers) than Level 1 or 2 survivors. However only eight survivors were classified to Level 1 and the survivors were aged only 16–39 years at survey (Absolom *et al*, 2006; Eiser *et al*, 2006).

The final of the three previous studies, including 112, 5-year childhood cancer survivors aged 18–45 years who were recruited from a late effects clinic, found that Level 3 survivors, reported more late effects than survivors classified to Level 2. However the Level 3 survivors were older than the Level 2 survivors and there was only one survivor classified to Level 1 (Michel *et al*, 2009).

Follow-up care proposed by the NCSI for Levels 1 and 2 have substantially reduced frequencies of hospital attendance compared with Level 3 (NHS Improvement, 2011a, 2011b; Glaser *et al*, 2013). Therefore it was essential to investigate whether those individuals proposed to be followed-up at Levels 1 or 2 experience an increased risk of any serious adverse health-outcomes which could potentially be avoided, or at least diagnosed at an earlier stage, with appropriate hospital follow-up. Detailed investigation for such evidence revealed that the NCSI flexible system of adjusting the level of care depending on existing morbidities is unlikely to lead to deaths which might be prevented or delayed (data not published due to the potentially individually identifiable nature of the data).

Strengths of our study include its large-scale and population-based design; also it benefits from substantially longer follow-up than available to any related previous study. In addition we have taken into account all serious adverse health-outcomes, including both those which were fatal or non-fatal, and so have avoided the limitation of previous related studies which were based on survivors alone. The population-based design of our study ensures that the observed and expected numbers, underlying the SMRs and SIRs, both relate to the entire population of Britain and avoid the potential biases relating to hospital-based studies. An additional strength of our study was that we had sufficient numbers to explore risk of adverse health outcomes by specific types of childhood cancer stratified by NCSI Levels of care. Such risk should be of practical benefit in clinics and for further developing standardised clinical follow-up guidelines (UK CCSG, 2005; SIGN, 2013; COG, 2013).

The principal weakness of our study relates to the crudeness of the cancer treatment information and the substantial fraction for whom treatment information was missing. However when sufficient numbers were available we have explored risks for specific FPN types who received cancer treatments of increasing levels of long-term toxicity by NCSI Levels 1, 2 and 3. Also our investigation of the potential impact of missing treatment on our risk estimates was mostly reassuring (Supplementary Appendices 3 and 4) and therefore it is unlikely that missing treatment has impacted the generalisability of our findings to the British population of childhood cancer survivors. Classification to NCSI Level used only limited information in the BCCSS computer record, before assessing available medical records. On subsequent detailed examination of their medical records we found that we inevitably classified some individuals initially to Level 1 or 2, when they should have been Level 3. However, since we reviewed the medical records of individuals contributing to each cause of death which was in excess of expected among Levels 1 and 2 survivors much of this misclassification was identified by the physicians. General population rates for England and Wales were used to generate expected numbers for SIRs and SMRs but excluding Scotland is unlikely to have important impact.

## Conclusion

The proposed NCSI risk stratification tool is simple, giving a considerable advantage in terms of clinical application. However, it provides clear and strong discrimination between survivors assigned to the three different levels in terms of their long-term cumulative risk of serious adverse health-outcomes in an appropriate rank order. It also provides clear and strong discrimination between survivors assigned to the three levels in relation to excess risks. This is reassuring as this tool is already in increasingly widespread use within the NHS for childhood cancer survivors.

As the risk stratification tool provides strong discrimination between groups of survivors, in terms of their long-term risk of adverse health outcomes, it is likely to be useful internationally. Furthermore with such strong discrimination, the survivor strata identified provide a basis for intervention studies of the various elements which comprise models of care, with a full economic evaluation, within a wide variety of health care systems whether privately or state organised.

## Figures and Tables

**Table 1:** Observed and expected numbers, SIRs and cumulative risks of specific types of subsequent primary neoplasms for survivors of non-leukaemic childhood cancers assigned to NCSI Levels 1, 2 and 3

	**LEVEL 1**				**LEVEL 2**				**LEVEL 3**			
**Specific types of second primary neoplasms**	**Obs**	**Exp**	**SIR (95% CI)**	**Cum. risk (95% CI)**^a^	**Obs**	**Exp**	**SIR (95% CI)**	**Cum. risk (95% CI)**^a^	**Obs**	**Exp**	**SIR (95% CI)**^b^	**Cum. risk (95% CI)**^a^, ^c^
Oral cavity and pharynx	0	0.4	N/A	N/A	7	1.0	7.2 (3.4–15.0)	0.49% (0.19–1.12%)	8	0.9	9.0 (4.5–17.9)*	0.31% (0.13–0.68%)
Digestive and peritoneum	4	1.8	2.2 (0.8–5.8)	0.53% (0.14–1.54%)	23	4.5	5.1 (3.4–7.7)	1.81% (1.06–2.92%)	38	3.8	10.0 (7.3–13.7)***(***)	2.59% (1.63–3.92%)^‡‡^(^‡‡‡^)
Colorectal	2	1.0	2.0 (0.5–7.9)	0.39% (0.07–1.42%)	13	2.5	5.2 (3.0–9.0)	1.12% (0.56–2.03%)	15	2.1	7.3 (4.4–12.0)	0.90% (0.45–1.63%)
Other digestive	2	0.8	2.5 (0.6–9.8)	0.14% (0.01–0.75%)	10	2.0	5.0 (2.7–9.3)	0.70% (0.28–1.51%)	23	1.7	13.2 (8.8–19.9)**(***)	1.70% (0.91–2.91%)^‡‡^(^‡‡‡^)
Respiratory and intrathoracic	2	1.0	2.0 (0.5–7.8)	0.38% (0.07–1.43%)	7	2.6	2.7 (1.3–5.6)	0.27% (0.09–0.68%)	9	2.2	4.1 (2.2–7.9)	1.33% (0.53–2.84%)
Bone and articular cartilage	6	0.2	24.7 (11.1–54.9)	0.39% (0.15–0.88%)	24	0.6	42.6 (28.6–63.6)	0.72% (0.46–1.07%)	42	0.6	65.3 (48.2–88.3)*(**)	1.32% (0.85–1.97%)^‡^(^‡‡^)
Melanoma	2	1.2	1.6 (0.4–6.5)	0.22% (0.05–0.76%)	9	2.7	3.3 (1.7–6.3)	0.47% (0.18–1.04%)	8	2.6	3.1 (1.5–6.1)	0.42 (0.17–0.94%)
NMSC	4	2.4	1.7 (0.6–4.5)	0.49% (0.15–1.28%)	41	5.6	7.4 (5.4–10.0)	3.12% (2.04–4.55%)	69	4.8	14.4 (11.4–18.2)***(***)	3.78% (2.63–5.25%)^‡‡‡^(^‡‡‡^)
Connective and soft tissue	1	0.3	3.4 (0.5–23.8)	0.08% (0.01–0.42%)	9	0.7	13.2 (6.9–25.3)	0.42% (0.16–0.94%)	26	0.7	37.0 (25.2–54.3)***(***)	1.66% (0.83–2.98%)^‡‡‡^(^‡‡‡^)
Breast	4	4.1	1.0 (0.4–2.6)	0.27% (0.08–0.77%)	16	9.5	1.7 (1.0–2.8)	1.35% (0.70–2.40%)	31	6.3	4.9 (3.4–7.0)***(***)	1.79% (1.08–2.79%)^‡‡^(^‡‡^)
Thyroid	1	0.4	2.8 (0.4–20.1)	0.32% (0.03–1.70%)	9	0.8	11.9 (6.2–22.9)	0.65% (0.30–1.28%)	18	0.7	24.2 (15.3–38.5)**(**)	0.61% (0.36–1.00%)^‡^(^‡‡^)
Genito-urinary	6	4.3	1.4 (0.6–3.1)	1.16% (0.30–3.26%)	20	10.1	2.0 (1.3–3.1)	1.77% (0.92–3.11%)	21	9.2	2.3 (1.5–3.5)	0.91% (0.43–1.76%)
Glioma CNS neoplasm	7	0.8	9.1 (4.4–19.2)	0.65% (0.28–1.35%)	12	1.8	6.8 (3.9–12.0)	0.35% (0.17–0.66%)	29	1.8	16.0 (11.2–23.1)*(*)	1.34% (0.75–2.24%)^‡^
NGCNS neoplasm	3	0.8	3.7 (1.2–11.6)	0.22% (0.04–0.79%)	18	1.8	9.9 (6.3–15.8)	1.27% (0.62–2.34%)	79	1.8	43.9 (35.2–54.7)***(***)	4.36% (3.02–6.06%)^‡‡‡^(^‡‡‡^)
Other & unspecified	7	5.0	1.4 (0.7–3.0)	0.41% (0.16–0.93%)	26	11.6	2.2 (1.5–3.3)	1.56% (0.84–2.67%)	15	12.0	1.3 (0.8–2.1)	0.61% (0.26–1.30%)^‡^
Total excluding NMSC	43	20.4	2.1 (1.6–2.9)	4.63% (2.96–6.84%)	180	47.6	3.8 (3.3–4.4)	11.13% (9.06–13.43%)	324	42.7	7.6 (6.8–8.5)***(***)	17.27% (14.62–20.11%)^‡‡‡^(^‡‡‡^)
Total excluding NGCNS	44	19.6	2.3 (1.7–3.0)	4.90% (3.19–7.14%)	203	45.8	4.4 (3.9–5.1)	12.63% (10.48–15.00%)	314	40.9	7.7 (6.9–8.6)***(***)	16.69% (14.14–19.43%)^‡‡‡^(^‡‡‡^)
Total excluding both NMSC and NGCNS	40	19.6	2.0 (1.5–2.8)	4.41% (2.77–6.62%)	162	45.8	3.5 (3.0–4.1)	10.30% (8.32–12.53%)	245	40.9	6.0 (5.3–6.8)***(***)	13.70% (11.31–16.32%)^‡‡‡^(^‡‡‡^)
Total	47	22.8	2.1 (1.6–2.8)	5.12% (3.38–7.38%)	221	53.2	4.2 (3.6–4.7)	14.25% (11.88–16.82%)	393	47.5	8.3 (7.5–9.1)***(***)	21.06% (18.16–24.10%)^‡‡‡^(^‡‡‡^)

Abbreviations: CI=confidence interval; CNS=central nervous system; Cum=cumulative; Exp=expected numbers; N/A=not applicable; NGCNS=Non-glioma CNS tumour; NMSC=non-melanoma skin cancer; Obs=total observed numbers; SIR=standardised incidence ratio.

a The reported cumulative incidences were at 45 years post diagnosis.

b From the test for heterogeneity for the respective SIR across the three different follow-up levels *P<0.05; **P<0.01; ***P<0.001. The parenthesis asterisks are from the test for trend for the SIRs across the three levels *P<0.05; **P<0.01; ***P<0.001.

c From the log-rank test for equality of survivor functions across the three levels ^‡^*P*<0.05; ^‡‡^*P*<0.01; ^‡‡‡^*P*<0.001. The parenthesis asterisks are from the log-rank test for trend of survivor functions across the three levels ^‡^*P*<0.05; ^‡‡^*P*<0.01; ^‡‡‡^*P*<0.001.

**Table 2:** Observed and expected numbers, SMRs and cumulative risks of specific non-neoplastic causes of death (upper half). Cumulative risks for corresponding specific non-fatal non-neoplastic conditions (lower half) for survivors of non-leukaemic childhood cancers assigned to NCSI Levels 1, 2 and 3

	**FATAL**											
	**LEVEL 1**				**LEVEL 2**				**LEVEL 3**			
**Specific non-neoplastic condition**	**Obs**	**Exp**	**SMR (95% CI)**	**Cum. risk (95% CI)**^a^	**Obs**	**Exp**	**SMR (95% CI)**	**Cum. risk (95% CI)**^a^	**Obs**	**Exp**	**SMR (95% CI)**^c^	**Cum. risk (95% CI)**^a^, ^d^
Circulatory	6	3.9	1.6 (0.7–3.5)	0.9% (0.3–2.2%)	36	9.5	3.8 (2.7–5.2)	2.1% (1.4–3.2%)	48	8.8	5.5 (4.1–7.2)^‡‡(‡‡)^	2.8% (1.8–4.3%)**(**)
Cardiac disease	5	2.7	1.8 (0.8–4.4)	0.8% (0.2–2.2%)	24	6.8	3.5 (2.4–5.2)	1.5% (0.8–2.4%)	25	6.4	3.9 (2.7–5.8)	1.4% (0.7–2.5%)
Cerebrovascular disease	1	0.9	1.2 (0.2–8.3)	0.1% (0.0–0.4%)	9	2.1	4.3 (2.3–8.4)	0.6% (0.2–1.2%)	20	1.9	10.8 (7.0–16.8)^‡‡(‡‡‡)^	1.3% (0.6–2.5%)**(**)
Pulmonary	6	1.2	4.9 (2.2–10.8)	1.0% (0.3–3.0%)	13	3.0	4.4 (2.6–7.6)	0.7% (0.3–1.4%)	35	2.9	12.3 (8.8–17.1)^‡‡(‡‡)^	2.5% (1.5–3.9%)**(**)
Gastro-intestinal	1	1.1	0.9 (0.1–6.4)	0.1% (0.0–0.4%)	4	2.6	1.6 (0.6–4.2)	0.3% (0.1–1.1%)	8	2.3	3.4 (1.7–6.9)	0.5% (0.2–1.1%)
Renal	1	0.2	5.0 (0.7–35.1)	0.1% (0.0–0.4%)	2	0.5	4.2 (1.0–16.7)	0.1% (0.0–0.5%)	6	0.4	13.5 (6.1–30.0)	0.2% (0.1–0.6%)
Musclo-skeletal	0	0.1	N/A	0.0% (N/A)	0	0.3	N/A	0.0% (N/A)	2	0.3	7.1 (1.8–28.3)	0.1% (0.0–0.6%)
Neurological	0	1.1	N/A	0.0% (N/A)	10	2.7	3.8 (2.0–7.0)	0.8% (0.3–1.9%)	23	2.8	8.3 (5.5–12.5)^‡‡‡(‡‡‡)^	1.6% (0.8–2.9%)**(***)
Endocrine	3	0.6	5.5 (1.8–16.9)	0.3% (0.1–0.8%)	3	1.3	2.3 (0.8–7.2)	0.1% (0.0–0.6%)	9	1.3	6.7 (3.5–12.9)	0.4% (0.2–1.0%)
Total above specified causes	17	8.2	2.1 (1.3–3.3)	2.3% (1.1–4.4%)	68	19.8	3.4 (2.7–4.4)	4.2% (3.0–5.7%)	131	18.8	7.0 (5.9–8.3)^‡‡‡(‡‡‡)^	8.2% (6.3–10.5%)***(***)
Other non-neoplastic causes^b^	6	10.6	0.6 (0.3–1.3)	0.7% (0.3–1.4%)	44	25.7	1.7 (1.3–2.3)	2.2% (1.5–3.2%)	46	28.8	1.6 (1.2–2.1)^‡^	1.7% (1.2–2.3%)*(*)

	**NON-FATAL**					
	**LEVEL 1**		**LEVEL 2**		**LEVEL 3**	
	**Obs**	**Cum. risk (95% CI)**^a^	**Obs**	**Cum. risk (95% CI)**^a^	**Obs**	**Cum. risk (95% CI)**^a^, ^d^
Vision	7	1.3% (0.6–2.9%)	21	1.6% (1.0–2.6%)	52	7.1% (4.7–10.7%)***(***)
Hearing	9	1.3% (0.7–2.5%)	53	4.8% (3.5–6.5%)	69	6.7% (4.6–9.7%)**(***)
Circulatory	6	2.4% (1.0–5.7%)	57	6.8% (4.7–9.9%)	102	12.4% (9.5–16.2%)***(***)
Pulmonary	9	2.4% (1.2–4.7%)	49	6.5% (4.5–9.3%)	61	5.4% (4.0–7.3%)**(***)
Gastro-intestinal	15	3.1% (1.6–5.7%)	36	4.3% (2.9–6.5%)	27	2.4% (1.3–4.3%)
Renal	6	0.8% (0.3–2.0%)	16	1.2% (0.7–2.2%)	36	4.5% (2.4–8.1%)**(**)
Musculo-skeletal	2	1.7% (0.4–7.0%)	27	3.6% (2.2–6.1%)	16	1.8% (0.7–5.1%)*
Neurological	2	0.3% (0.1–1.0%)	18	2.3% (1.2–4.2%)	48	4.1% (2.7–6.4%)***(***)
Endocrine	12	2.9% (1.3–6.5%)	52	4.2% (3.0–5.7%)	101	7.8% (5.7–10.5%)***(***)
For any of the specified conditions	59	13.8% (9.9–19.1%)	278	27.3% (23.7–31.3%)	423	39.9% (35.1–45.0%)***(***)

Abbreviations: CI=confidence interval; Cum=cumulative; Exp=expected numbers; N/A=not applicable; Obs=total observed numbers; SMR=standardised mortality ratio.

a The reported cumulative incidences were at 45 years post diagnosis.

b This includes deaths from infections, blood disease, mental disorders, pregnancy and childbirth, external causes and others.

c From the test for heterogeneity for the respective SMR across the three different follow-up levels ^‡^*P*<0.05; ^‡‡^*P*<0.01; ^‡‡‡^*P*<0.001. The parenthesis asterisks are from the test for trend for the respective SMR across the three different follow-up levels ^‡^*P*<0.05; ^‡‡^*P*<0.01; ^‡‡‡^*P*<0.001.

d From the log-rank test for equality of survivor functions across the three levels *P<0.05; **P<0.01; ***P<0.001. The parenthesis asterisks are from the log-rank test for trend of survivor functions across the three levels *P<0.05; **P<0.01; ***P<0.001.

**Table 3:** Cumulative risk (excess risk) of specified adverse health outcomes by 45 years from diagnosis of all childhood cancers combined except leukaemia for specific NCSI Levels

	**NCSI Level of Clinical Care**		
**Adverse health outcome**	**1**	**2**	**3**
Any subsequent primary neoplasm	5% (2-fold)	14% (4-fold)	21% (8-fold)
Any potentially treatment related non-neoplastic death	2% (2-fold)	4% (3-fold)	8% (7-fold)
Any potentially treatment related non-fatal non-neoplastic condition	14%	27%	40%
Overall cumulative risk	21%	45%	69%
