# Supplementary material for: Risk stratification of childhood cancer survivors necessary for evidence-based clinical long-term follow-up
Source: Br J Cancer. 2017 Oct 24;117(11):1723–31. doi: 10.1038/bjc.2017.347 (PMC5729444; doi:10.1038/bjc.2017.347)
Supplement: Supplementary Information [file bjc2017347x1.docx]

**Appendix 1**

Details on the levels of care. Taken from the report “Models of care to achieve better outcomes for children and young people living with and beyond cancer”. NHS Improvement 2011.

<http://webarchive.nationalarchives.gov.uk/20130221101407/http://www.improvement.nhs.uk/LinkClick.aspx?fileticket=Y1CGhGEoXsg%3d&tabid=56>

**Appendix 2**

**Appendix Table 1: Cumulative risks^#^ and SIRs of any SPN*, cumulative risks^#^ and SMRs of any fatal non-neoplastic condition, and cumulative risks^#^ of any non-fatal non-neoplastic condition among Level 1, 2 and 3 survivors of specific childhood cancers, for which there was at least 100 survivors still alive 25 years‡ from diagnosis.**

| **FPN type** | **LEVEL 1** | | **LEVEL 2** | | **LEVEL 3** | |
| --- | --- | --- | --- | --- | --- | --- |
|  | **SPN** | | | | | |
|  | **Cum. risk (95% CI)#** | **SIR (95% CI)** | **Cum. risk (95% CI)#** | **SIR (95% CI)** | **Cum. risk (95% CI)#** | **SIR (95% CI)** |
| CNS neoplasm |  |  | 1.9% (1.1 to 3.0%) | 1.9 (1.3 to 2.8) | 2.7% (2.0 to 3.6%) | 3.4 (2.7 to 4.3) |
| Hodgkin's lymphoma |  |  |  |  | 6.1% (4.4 to 8.2%) | 6.4 (4.9 to 8.2) |
| Non-Hodgkin's lymphoma |  |  | 3.7% (2.1 to 6.0%) | 4.3 (2.9 to 6.3) |  |  |
| Neuroblastoma |  |  | 1.5% (0.4 to 4.2%) | 5.1 (2.9 to 8.9) |  |  |
| Non-heritable retinoblastoma | 0.8% (0.2 to 2.6%) | 1.3 (0.6 to 2.8) |  |  |  |  |
| Heritable retinoblastoma |  |  |  |  | 12.9% (9.1 to 17.3%) | 16.8 (13.2 to 21.2) |
| Wilms' tumour |  |  | 2.4% (1.0 to 4.8%) | 4.6 (3.0 to 7.0) | 3.6% (2.3 to 5.5%) | 6.5 (4.4 to 9.6) |
| Bone sarcoma |  |  |  |  |  |  |
| Soft tissue sarcoma | 0.6% (0.1 to 2.9%) | 1.9 (1.0 to 3.8) | 3.5% (1.7 to 6.3%) | 3.1 (1.8 to 5.2) |  |  |
| Acute lymphoblastic leukaemia |  |  | 1.1% (0.5 to 2.2%) | 2.7 (1.4 to 5.4) | 1.5% (0.9 to 2.4%) | 5.8 (3.9 to 8.5) |
|  | **FATAL NON-NEOPLASTIC CONDITION** | | | | | |
|  | **Cum. risk (95% CI)#** | **SMR (95% CI)** | **Cum. risk (95% CI)#** | **SMR (95% CI)** | **Cum. risk (95% CI)#** | **SMR (95% CI)** |
| CNS neoplasm |  |  | 1.4% (0.8 to 2.4%) | 3.1 (1.9 to 4.9) | 2.9% (2.1 to 3.8%) | 9.1 (7.4 to 11.2) |
| Hodgkin's lymphoma |  |  |  |  | 2.2% (1.2 to 3.6%) | 4.9 (3.3 to 7.4) |
| Non-Hodgkin's lymphoma |  |  | 1.9% (0.9 to 3.4%) | 5.0 (3.0 to 8.3) |  |  |
| Neuroblastoma |  |  | 2.1% (0.7 to 5.0%) | 5.6 (2.5 to 12.4) |  |  |
| Non-heritable retinoblastoma | 0.4% (0.0 to 2.0%) | 0.5 (0.1 to 3.6) |  |  |  |  |
| Heritable retinoblastoma |  |  |  |  | 0.8% (0.2 to 2.5%) | 2.3 (0.8 to 6.0) |
| Wilms' tumour |  |  | 0.0% | 0.5 (0.1 to 3.5) | 0.8% (0.3 to 1.9%) | 4.7 (2.2 to 9.8) |
| Bone sarcoma |  |  | 0.5% (0.1 to 2.7%) | 1.4 (0.3 to 5.5) |  |  |
| Soft tissue sarcoma | 0.8% (0.2 to 2.6%) | 1.0 (0.2 to 3.9) | 0.8% (0.2 to 2.7%) | 3.3 (1.6 to 7.0) |  |  |
| Acute lymphoblastic leukaemia |  |  | 0.1% (0.01 to 0.5%) | 0.9 (0.1 to 6.3) | 1.0% (0.5 to 1.7%) | 8.0 (4.6 to 13.8) |
|  | **NON-FATAL NON-NEOPLASTIC CONDITION** | | | | | |
|  | **Cum. risk (95% CI)#** |  | **Cum. risk (95% CI)#** |  | **Cum. risk (95% CI)#** |  |
| CNS neoplasm |  |  | 11.7% (9.3 to 14.8%) |  | 18.6% (16.1 to 21.5%) |  |
| Hodgkin's lymphoma |  |  |  |  | 15.2% (11.6 to 19.8%) |  |
| Non-Hodgkin's lymphoma |  |  | 9.0% (5.8 to 13.7%) |  |  |  |
| Neuroblastoma |  |  |  |  |  |  |
| Non-heritable retinoblastoma | 5.1% (2.6 to 9.9%) |  |  |  |  |  |
| Heritable retinoblastoma |  |  |  |  | 7.1% (3.8 to 13.3%) |  |
| Wilms' tumour |  |  | 8.6% (5.3 to 13.9%) |  | 15.7% (12.3 to 20.0%) |  |
| Bone sarcoma |  |  |  |  |  |  |
| Soft tissue sarcoma |  |  | 4.5% (2.2 to 9.3%) |  |  |  |
| Acute lymphoblastic leukaemia |  |  | 4.5% (2.8 to 7.1%) |  | 9.2% (7.0 to 12.0%) |  |

ALL - Acute lymphoblastic leukaemia; CI -confidence interval; CNS - central nervous system; FPN - first primary neoplasm; N/A - not applicable; SIR - standardised incidence ratio; SMN - subsequent malignant neoplasm; SMR - standardised mortality ratio.

# - Cumulative risks by 25 years from diagnosis, except after ALL which is by 20 years from diagnosis.

‡ - Except for ALL which is at 20 years post diagnosis.

* - The SMNs exclude NGCNS tumours and NMSC.

**Appendix 3**

**Flow chart of included/excluded individuals.**

**All childhood cancer survivors except leukaemia**

Population-based registry data - SPNs & deaths Questionnaire data - Non-fatal non-neoplastic outcomes

Level 1

n = 1,358

Died before contact

n = 1,979

Eligible for questionnaire

n = 10,865

Level 2

n = 3,337

Whole BCCSS cohort n=13,130

Whole BCCSS cohort n=13,130

Level 3

n = 3,980

Alive but aged under <16y at contact; embarked; not registered with GP; adopted: n = 286

No questionnaire returned n = 3,255

Questionnaire completed and returned n = 7,610

No missing treatment information n = 4,960

Level 1

n = 777

Level 2

n = 2,032

Level 3

n = 2,151

No missing treatment information n = 8,675

Appendix Figure 1: Comparison of cumulative incidences for those with missing treatment information and those with no missing treatment information, for all childhood cancer survivors except leukaemia, for any subsequent primary neoplasm.

Log rank test for equality of observed risks yields p = 0.0003

Appendix Figure 2: Comparison of cumulative incidences for those with missing treatment information and those with no missing treatment information, for all childhood cancer survivors except leukaemia, for any fatal non-neoplastic outcome

Log rank test for equality of observed risks yields p = 0.2965

Appendix Figure 3: Comparison of cumulative incidences for those with missing treatment information and those with no missing treatment information, for all childhood cancer survivors except leukaemia, for any non-fatal non-neoplastic outcome.

Log rank test for equality of observed risks yields p = 0.0947

**Appendix 4**

**Flow chart of included/excluded individuals.**

**Survivors of acute lymphoblastic leukaemia**

Population-based registry data – SPNs & deaths Questionnaire data - Non-fatal non-neoplastic outcomes

Level 1

n = 190

Included in a UKALL trial n=2,844

Died before contact

n = 390

Questionnaire completed and returned n = 1,754

No missing treatment information n = 1,615

Level 3

n = 694

Level 2

n = 781

Level 1

n = 140

Included in a UKALL trial n=2,844

Level 2

n = 1,106

No missing treatment information n = 2,638

Whole BCCSS cohort n=4,408

Whole BCCSS cohort n=4,408

Level 3

n = 1,342

Non-response to questionnaire; alive but aged under <16y at contact; not registered with clinician: n = 700

N.B. AML, CML and other non-ALL leukaemias n = 443 are excluded as there are insufficient for meaningful separate analysis.

Appendix Figure 4: Comparison of cumulative incidences for those with missing treatment information and those with no missing treatment information, for survivors of acute lymphoblastic leukaemia, for any subsequent primary neoplasm.

Log rank test for equality of observed risks yields p = 0.9139

Appendix Figure 5: Comparison of cumulative incidences for those with missing treatment information and those with no missing treatment information, for survivors of acute lymphoblastic leukaemia, for any fatal non-neoplastic outcome.

Log rank test for equality of observed risks yields p = 0.4999

Appendix Figure 6: Comparison of cumulative incidences for those with missing treatment information and those with no missing treatment information, for survivors of acute lymphoblastic leukaemia, for any non-fatal non-neoplastic outcome.

Log rank test for equality of observed risks yields p = 0.6890
